# Use of a novel electronic maternal surveillance system to generate automated alerts on the labor and delivery unit

**DOI:** 10.1186/s12871-018-0540-6

**Published:** 2018-06-26

**Authors:** Thomas T. Klumpner, Joanna A. Kountanis, Elizabeth S. Langen, Roger D. Smith, Kevin K. Tremper

**Affiliations:** 1 Department of Anesthesiology, University of Michigan, 1H247 University Hospital, 1500 East Medical Center Drive, Ann Arbor, MI 48109-5048 USA; 2 Department of Obstetrics and Gynecology, Von Voigtlander Women’s Hospital, University of Michigan, Ann Arbor, MI USA

**Keywords:** Obstetrics, Obstetrical anesthesia, Clinical decision support systems

## Abstract

**Background:**

Maternal early warning systems reduce maternal morbidity. We developed an electronic maternal surveillance system capable of visually summarizing the labor and delivery census and identifying changes in clinical status. Automatic page alerts to clinical providers, using an algorithm developed at our institution, were incorporated in an effort to improve early detection of maternal morbidity. We report the frequency of pages generated by the system. To our knowledge, this is the first time such a system has been used in peripartum care.

**Methods:**

Alert criteria were developed after review of maternal early warning systems, including the Maternal Early Warning Criteria (MEWC). Careful consideration was given to the frequency of pages generated by the surveillance system. MEWC notification criteria were liberalized and a paging algorithm was created that triggered paging alerts to first responders (nurses) and then managing services due to the assumption that paging all clinicians for each vital sign triggering MEWC would generate an inordinate number of pages. For preliminary analysis, to determine the effect of our automated paging algorithm on alerting frequency, the paging frequency of this system was compared to the frequency of vital signs meeting the Maternal Early Warning Criteria (MEWC). This retrospective analysis was limited to a sample of 34 patient rooms uniquely capable of storing every vital sign reported by the bedside monitor.

**Results:**

Over a 91-day period, from April 1 to July 1, 2017, surveillance was conducted from 64 monitored beds, and the obstetrics service received one automated page every 2.3 h. The most common triggers for alerts were for hypertension and tachycardia. For the subset of 34 patient rooms uniquely capable of real-time recording, one vital sign met the MEWC every 9.6 to 10.3 min. Anecdotally, the system was well-received.

**Conclusions:**

This novel electronic maternal surveillance system is designed to reduce cognitive bias and improve timely clinical recognition of maternal deterioration. The automated paging algorithm developed for this software dramatically reduces paging frequency compared to paging for isolated vital sign abnormalities alone. Long-term, prospective studies will be required to determine its impact on patient outcomes.

## Background

Maternal mortality has decreased globally but is on the rise in the United States [1]. Delays in diagnosis or in the initiation of treatment contribute to a large portion of pregnancy-related deaths [2, 3]. As a consequence, the National Partnership for Maternal Safety has encouraged hospitals to adopt the Maternal Early Warning Criteria (MEWC) to reduce delays in recognition and treatment of maternal morbidity [4]. These criteria comprise a list of abnormal maternal vital signs that call for an urgent bedside evaluation [5]. Vigilant bedside reassessment tied to maternal early warning systems, promotes early diagnosis and timely intervention, and reduces maternal morbidity [6].

Our institution implemented a maternal early warning system, based on the Maternal Early Warning Criteria, in 2013. Initial obstacles to implementation included logistic difficulties in paging multiple services, multidisciplinary collaboration, and cognitive bias leading to individual errors in judgment. These obstacles to implementation of early warning systems have been described previously [7, 8].

In order to bypass these obstacles, and at the request of our unit’s quality improvement committee, we designed AlertWatch™ OB at our institution. This is a novel electronic maternal surveillance and early warning system, designed to access vital sign monitoring and automatically alert clinicians to deviations from a predetermined acceptable range [4]. AlertWatch™ OB works in parallel with the existing maternal early warning system, acting as a “safety net” by automatically notifying providers about profound vital sign abnormalities as soon as they are first detected. While automated electronic surveillance systems have been used to facilitate intraoperative glucose management [9], recognize medication errors [10], and attempt to facilitate recognition and management of sepsis [11, 12], this is the first time that they have been used in peripartum care.

This manuscript describes an electronic maternal surveillance system, designed at the authors' institution that is designed to: 1) access patient vital sign monitoring and other clinical data, 2) present clinical status in a visual format for each individual patient and 3) present clinical summaries as a visual “patient census view” for the entire labor and delivery unit, and 4) alert staff of clinical deterioration. As a preliminary analysis, to determine the effect of the paging notification algorithm on paging frequency, the frequency of automated pages generated by this surveillance system was compared with the frequency of abnormal vital signs recorded by bedside monitors that met the Maternal Early Warning Criteria (MEWC) [4].

## Methods

The surveillance system is a secure web-based multifunction display that receives, integrates, and summarizes data from physiologic monitors, electronic health records, and laboratory systems. A prototype of the system was designed by two authors (TK and KT) and made available to all labor and delivery nurses, nurse midwives, obstetricians and anesthesiologists in June 2016. The design of the software and paging algorithm was finalized in April 2017 and electronic status boards running the software were installed in the anesthesiology and obstetrics team rooms.

**Fig. 1:**
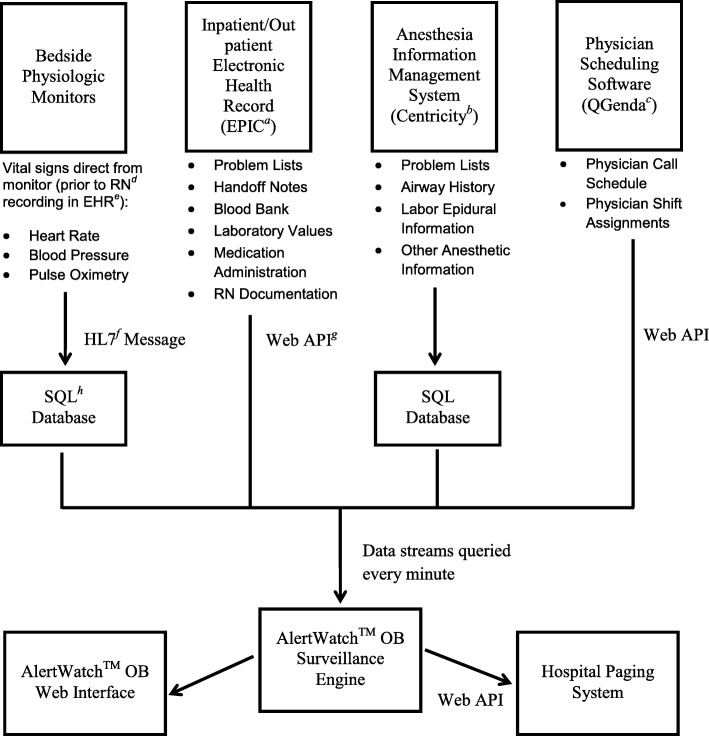
Data Flow Diagram. Information from bedside physiologic monitors, the electronic health record, the anesthesia information management system and the physician scheduling software are queried every minute by the surveillance engine. This aggregated data is then presented to clinicians via a web interface. Pages are sent to clinicians for the most severe changes in patient condition. ^a^MiChart, an implementation of the EPIC clinical information system (EPIC Systems Inc., Verona, WI), is used at our institution. ^b^The anesthesia information management system at our institution is an implementation of Centricity™ Perioperative Anesthesia (GE Healthcare, Chicago, IL). ^c^QGenda, QGenda, LLC, Atlanta, GA. ^d^RN = Registered Nurse. ^e^EHR = Electronic Health Record. ^f^HL7 = Health Level 7. ^g^API = Application Programming Interface. ^h^SQL = Structured Query Language

**Fig. 2:**
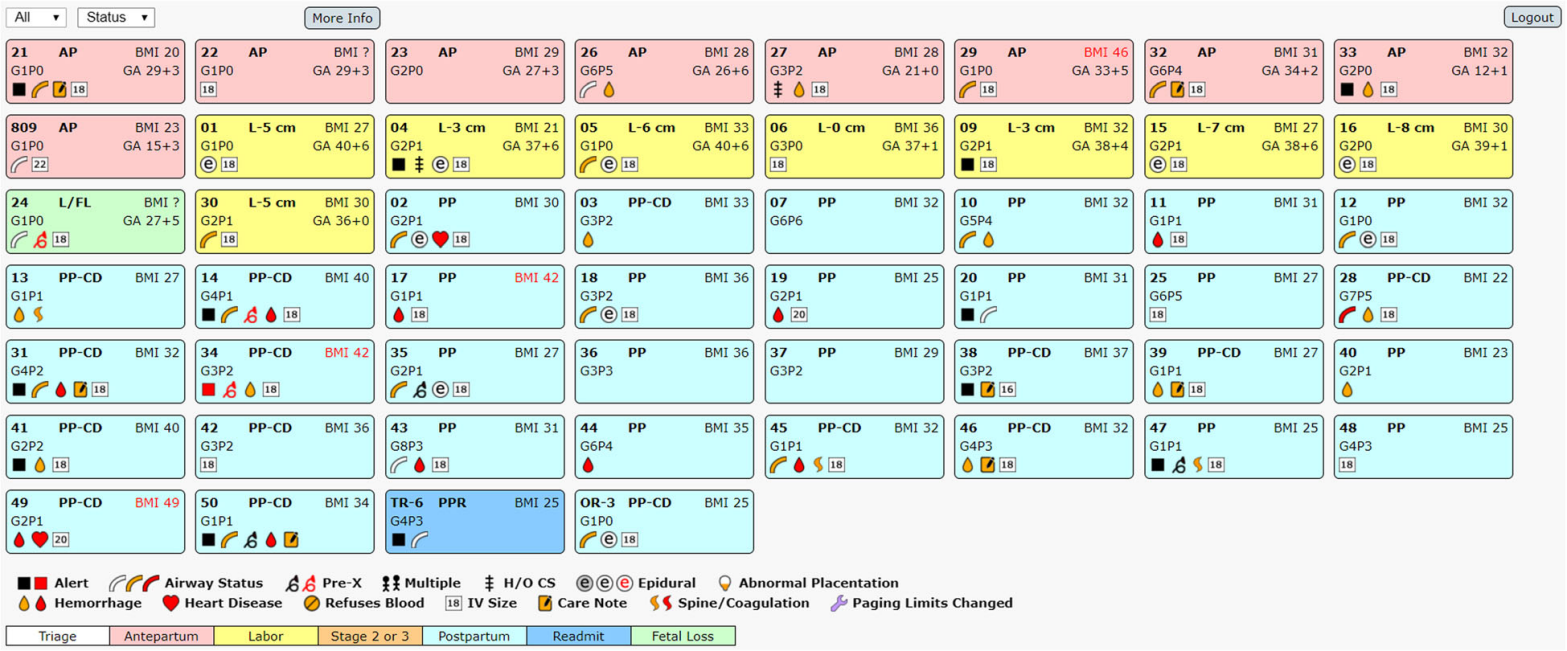
Patient Census View. Each patient on the labor and delivery census is represented by a rectangle with patient information displayed in a standardized fashion. These are organized into a grid to summarize the labor and delivery service. For example, there are 9 laboring patients on the unit, with one patient who experienced a fetal loss. The patient in room 4 is attempting a trial of labor after cesarean delivery and has an epidural in place with a pain score less than 4. The two patients with pertinent cardiac comorbidities have already delivered. One patient, in room 34, may require immediate evaluation

**Fig. 3:**
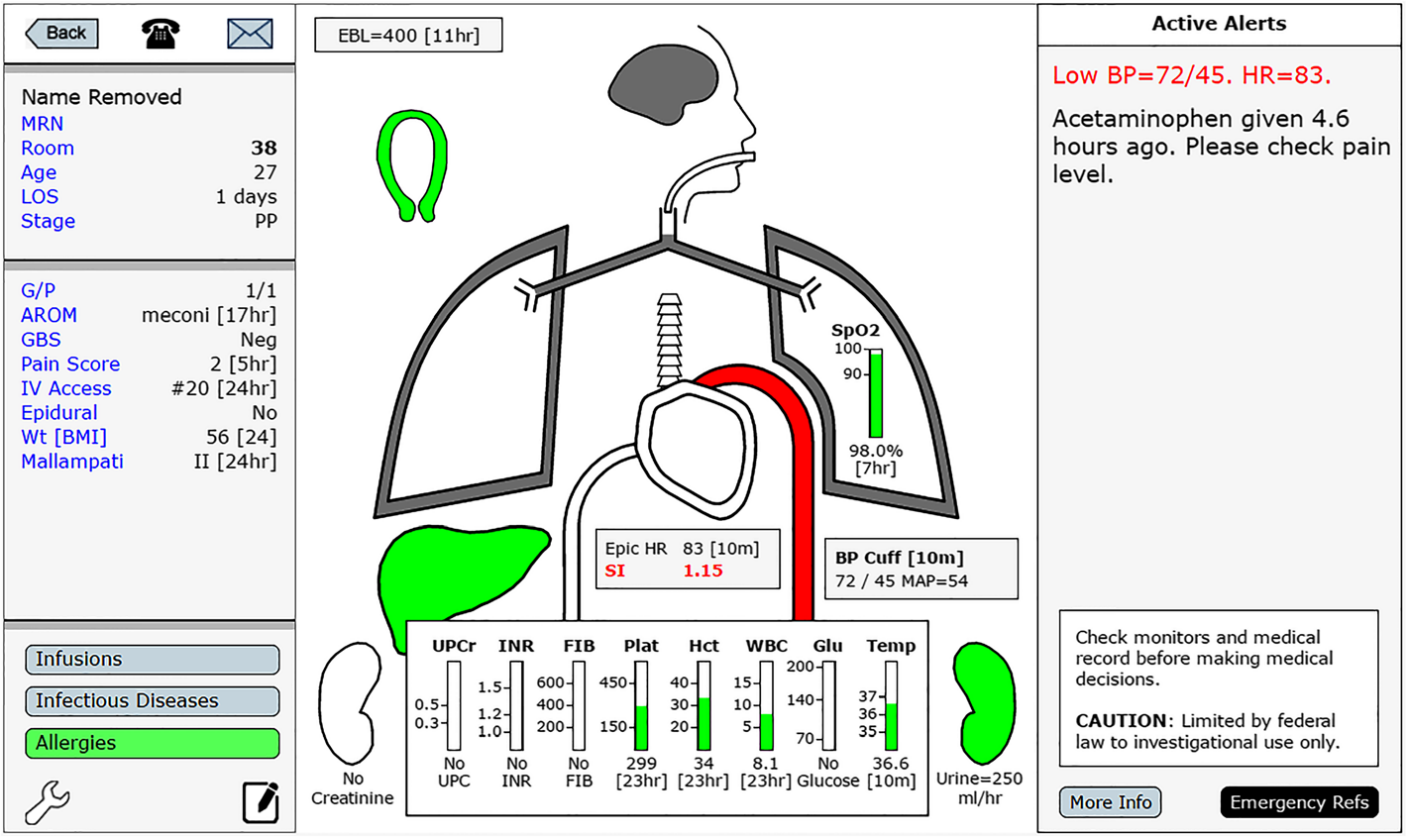
Individual Patient View. Any patient rectangle in the patient census can be selected for individual review. This individual patient display is comprised of readily identifiable icons of human organs, whose colors change as measured parameters change from normal to marginal to abnormal risk-adjusted ranges. Alerts are prominently displayed in the column on the right. In this example, the patient has a “flashing red” alert, with a blood pressure of 72/45 mmHg

The system is based on an Food and Drug Administration (FDA) cleared decision support multifunction display developed in our institution for general operating rooms, AlertWatch™ OR (AlertWatch, Inc., Ann Arbor, MI, USA) [13]. This system, AlertWatch™ OB, has also received FDA clearance. The software queries patient data streams every minute to obtain a near-real time feed of physiologic data and does not require additional provider charting or input of patient data. A data flow diagram is shown in Fig. 1. Unit information is summarized visually with a patient census, as shown in Fig. 2, using an icon system detailed in Table 1. Additionally, any individual patient can be examined in detail, as shown in Fig. 3.

**Table 1:** Icons Used in the Patient Surveillance View

Condition	Icon	Description	Data Source(s)
Airway Exam Missing		This icon is displayed when an airway examination has not been documented.	AIMS^*a*^
At Risk for Difficult Intubation		This patient is at risk for difficult intubation. This is defined by the documentation of three or more of the following risk factors: Mallampati score III, body mass index > 30, limited jaw protrusion, history of obstructive sleep apnea, history of snoring, presence of a thick neck or radiation changes.	AIMS
Known Difficult Intubation		This patient had prior documented difficulty with intubation. This is defined by the documentation of one of the following: Mallampati score IV, difficult airway letter, fiberoptic intubation, video laryngoscope intubation, difficulty with mask ventilation, Cormack-Lehane direct laryngoscopy view of three or four.	AIMS
Documentation Deficiency		The patient’s chart contains a documentation deficiency.	EHR^*b*^
Black Alert		Always displayed in the lower right hand corner of a patient rectangle if conditions for a black alert are met.	Bedside Monitor, EHR, Laboratory System, Blood Bank
Red Alert		Always displayed in the lower right hand corner of a patient rectangle if conditions for a red alert are met. This icon will flash if conditions for a flashing red alert are met.	Bedside Monitor, EHR, Laboratory System, Blood Bank
IV Size		IV size currently in place. The number within the box indicates the documented gauge of the IV.	EHR
Heart Disease		Displayed if the patient has any documented cardiac disease.	AIMS, EHR, Text Parser^*c*^
Refuses Blood		Displayed if documentation is found indicating patient refusal of blood products.	AIMS, EHR, Text Parser
Care Note		Displayed if a multidisciplinary care note is found for this patient. If present, this care note is found in a consistent location within the patient EHR.	EHR
Prior Cesarean Delivery or Uterine Surgery		Displayed if there is a documented history of cesarean delivery or diagnosis code related to surgery involving the uterus.	AIMS, EHR, Text Parser
Pre-eclampsia		Displayed with documentation of pre-eclampsia without severe features.	EHR, AIMS, Text Parser
Severe Pre-eclampsia		Displayed with documentation of pre-eclampsia with severe features. This is also displayed if a magnesium infusion has been documented within the EHR medication administration record.	EHR, AIMS, Text Parser
Multiple Gestation		Displayed if patient has a multiple gestation pregnancy.	EHR, AIMS, Text Parser
Epidural		An epidural catheter is in place. The icon turns red if the patient’s pain score ≥ 5. The icon disappears after documentation of removal of the epidural catheter.	AIMS, EHR
Abnormal Placentation		Displayed with documentation of abnormal placentation, i.e. placenta previa, placenta accreta, placenta increta, or placenta percreta.	AIMS, EHR, Text Parser
Risk of Postpartum Hemorrhage		Displayed if patient is suspected to be at risk for postpartum hemorrhage.	AIMS, EHR, Text Parser
Postpartum Hemorrhage		Displayed if > 500 mL of blood loss is documented after vaginal delivery, or if > 1000 mL of blood loss is documented after cesarean section.	AIMS, EHR
Spine/Anticoagulation Warning		Displayed if an absolute or relative contraindication to neuraxial anesthesia is present. E.g. therapeutic anticoagulation.	AIMS, EHR
Paging Limit Change		Displayed if the automated paging thresholds for this patient have been modified.	N/A^*d*^

^*a*^AIMS = Anesthesia Information Management System. The AIMS at our institution is an implementation of Centricity™ Perioperative Anesthesia (GE Healthcare, Chicago, IL). Unless otherwise specified, data are collected from discrete fields within the AIMS

^*b*^EHR = Inpatient Electronic Health Record. MiChart, an implementation of the EPIC clinical information system (EPIC Systems Inc., Verona, WI), is used at our institution. Unless otherwise specified, data are collected from International Classification of Diseases (ICD-9 or ICD-10) codes contained within the patient problem list and from discrete fields within the EHR

^*c*^Text Parser = A text parser was created to detect comorbidities documented within non-discrete fields within the AIMS history and physical document and within free-text handoff documentation in the EHR

^*d*^N/A = Not Applicable

Individual patient data are accessed from the labor and delivery unit, which serves approximately 4500 deliveries per year. Geographically this includes 30 labor and antepartum rooms, 20 postpartum rooms, 8 triage rooms, 4 operating rooms and 6 pre- and post-anesthesia care (PACU) bays. All rooms are monitored by the surveillance system simultaneously, with the exception of the operating rooms, which are monitored separately by AlertWatch™ OR.

This surveillance system is designed to draw attention to specific patients who may warrant additional evaluation with a customizable tiered visual and paging alerting system. These alerts, chosen after review of different maternal early warning systems [4, 6], are summarized in Table 2. Conditions in boldface type trigger a “flashing red” square icon alert on the census view and are tied to an automated page. The other conditions trigger a visual black or red alert on the patient census.

**Table 2:** Tiered Maternal Physiologic Alerts

Parameter	Low Red	Low Black	Normal	High Black	High Red	Notes
Systolic Blood Pressure^*a*^	**< 85** ^***b***^	85–89	90–139	140–159	**≥ 160**	
Diastolic Blood Pressure^*a*^		< 50	50–99	100–109	**≥ 110**	
Temperature^*c*^		< 36	< 38	≥ 38	≥ 38.5	
Heart Rate^*d*^		< 50		≥ 120	**≥ 130**	
Pulse oximetry	< 93%	< 95%	≥ 95%			
Oliguria	**< 30 ml/hr**					Alert is only triggered if a magnesium infusion is being administered.
Shock Index (heart rate divided by systolic blood pressure)				≥ 1.2	**≥ 1.3**	Alert will only trigger if systolic blood pressure is also < 105 mmHg, and patient is within 4 h of delivery.
Hematocrit	**< 18%**	< 21%				
Glucose^*e*^	< 50, < 60	< 70	70–149	150–279	≥ 280	Glucose < 70 mg/dL alerts only if patient is on insulin or in in the operating room. Glucose < 50 mg/dL creates a red alert for every patient.
Unmeasured Blood Glucose with Insulin Infusion				Blood glucose measured 2 hours ago.	Blood glucose measured 3 h ago.	
Platelet Count^*f*^		< 80				

^a^Units are millimeters of mercury

^b^Values in bold typeface are parameters that will trigger a “flashing red” alert, which will also generate a page to clinicians

^c^Units are degrees Centigrade

^d^Units are beats per minute

^e^Units are milligrams per deciliter

^f^Units are thousands per microliter

**Fig. 4:**
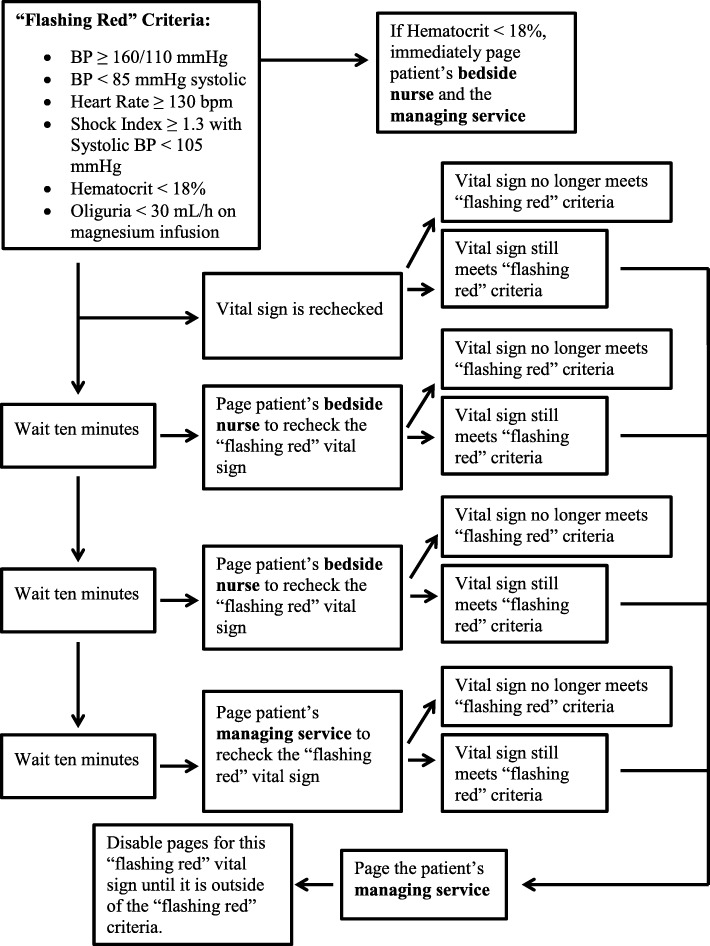
Automated Paging Sequence. Automatic pages are sent to a patient’s bedside nurse and managing service when defined “flashing red” criteria are met

When a “flashing red” alert occurs, an automated page is generated initially to the patient’s bedside nurse after a 10 min delay with a request to verify the finding. If the finding is verified (i.e. criteria for a “flashing red” alert are met for the same parameter), a second paging alert is sent automatically to physician services on the unit. If the vital sign is not verified within 30 min, an automatic page is sent to physician services on the unit with a request to verify the abnormal vital sign. The delay periods were chosen by consensus to reduce alarm fatigue by allowing time for a nurse to recognize abnormal vital signs unprompted and so act accordingly. The software also allows clinicians to adjust the paging parameters for individual patients. If laboratory data reveal severe anemia, a page is automatically sent without delay to the patient’s bedside nurse, the managing service, and the anesthesiology service. This tiered paging sequence is summarized in Fig. 4.

During its design, a paging log was created to track the actual paging frequency of the system. Careful consideration was given to the frequency of pages that were generated. It was assumed that frequent pages to clinicians on the labor and delivery unit, even if accurate, would result in alarm fatigue. It was also assumed that paging for every vital sign meeting predefined maternal early warning system criteria, such as those of the MEWC, would result in an inordinate number of pages across 64 patient rooms. Therefore, the MEWC criteria was liberalized, e.g. pages are generated for a heart rate in excess of 130 bpm instead of a heart rate above 120 bpm, to reduce paging frequency. Nurses were also used as first responders to confirm aberrant vital signs. Additionally, based on initial user feedback, pages were suspended during the second stage of labor when maternal expulsive efforts naturally generate tachycardia and hypertension. It is also assumed that during this time, a patient is being closely monitored as there is one to one nursing care with the nurse generally at the bedside during this time. Page alerts for hypoxemia (SpO2 <  95%) and bradycardia (HR <  50 bpm), were also eliminated during the early stages of implementation as the majority of our users felt these pages to be artefactual.

Institutional Review Board (IRB) approval was obtained to ascertain the actual paging frequency of the surveillance system and compare this to the frequency whereby abnormal vital signs met the MEWC criteria [4] in order to determine if the changes to MEWC notification criteria resulted in a meaningful reduction in pages to clinicians. This comparison allows the determination of a theoretical paging frequency if pages were generated for all vital signs meeting the MEWC, instead of the circumstances outlined in Fig. 4. This query was part of a larger ongoing IRB approved study. Written consent was not required by the University of Michigan IRB. This query used a database without direct patient identifiers and involved no more than minimal risk to the subjects. Additionally, completely accurate identification of individual patients for consent was not possible given the lack of patient identifiers in the original dataset.

For this analysis, data from a sample of 34 patient rooms with networked bedside monitors capable of real-time data extraction and recording were assessed. These rooms were chosen since a record of every single vital sign taken from the bedside monitor is stored in a separate database. These vital signs may be different from the vital signs documented in the electronic health record if, for example, the bedside nurse believes a set of vitals to be artefactual and takes the set of vitals a second time. In this situation, the electronic health record may only contain one set of vitals, while the monitor recording would contain two sets of vitals. Since the system’s paging algorithm uses unvalidated vital signs taken directly from a bedside monitor, we felt that the unique recording capabilities of these 34 rooms would give the most accurate retrospective assessment of theoretical changes to our paging algorithm. These rooms are preferentially used in the care of high risk patients because of these networked monitors and proximity to nursing stations, however they are also often used in the care of low risk patients. These rooms are used for antepartum, intrapartum and postpartum care. Vital signs may be taken every 15 min for high risk patients, depending on the severity of the patient condition. If any room is used for a low risk patient, vital signs may be taken every 8 h.

The maternal surveillance system was fully implemented in April 2017. Data were analyzed over a 91 day period from April 1, 2017 to July 1, 2017. Vital signs recorded from the bedside monitors in the 34 room sample were extracted and the frequency of vital signs meeting the MEWC were determined. The paging logs of the surveillance system were also queried. Pages generated by the surveillance system over the study period for the entire unit and for the sample of 34 rooms were determined.

## Results

Over a 91 day period, from April 1, 2017 to July 1, 2017, across 64 monitored rooms, the obstetric medical staff received 947 automated pager alerts, averaging one page every 2.3 h. The bedside nursing staff received 1180 automated physiologic pages. The average nurse caring for two patient rooms received approximately one automated pager alert every 59.3 h. The most common pager alerts were for hypertension (47.6%) and tachycardia (28.2%).

Over the same 91 day period, the bedside vital sign monitors in the sample of 34 patient rooms recorded 47,016 blood pressure (BP) measurements, 97,558 pulse oximeter measurements (SpO2) and 143,799 heart rates (see Table 3). These measurements were automatically captured by the monitor and not verified by the bedside nurse. The most frequent vital sign abnormality recorded by these bedside monitors was an SpO2 reading less than 95%. Of these measurements, 4253 met the “flashing red” criteria, and 13,671 met the MEWC. Therefore, an average of 46.7 readings per day (one every 30.8 min) met the “flashing red” criteria and 150.2 readings per day (one every 9.6 min) met the MEWC. If one conservatively assumes that all hypertensive readings were accompanied by both systolic and diastolic hypertension, an average of 41.4 readings per day met the “flashing red” criteria (one every 34.8 min) and 140.2 readings per day (one every 10.3 min) met the MEWC. Therefore, if these 34 bedside monitors were programmed to page a clinician for every value meeting the MEWC, clinicians would have received one page every 9.6 to 10.3 min.

**Table 3:** Frequency of Aberrant Vital Signs Recorded by High Risk Bedside Monitors

Number of Recorded Values^*a*^	Maternal Early Warning Criteria				Authors’ “Flashing Red” Criteria				Authors’ Automated Paging Criteria		
	Criteria	Events^*b*^	Percent of Recorded Values	Events/24 Hours	Criteria	Events	Percent of Recorded Values	Events/24 Hours	Physician Paging Criteria	Pages to OB	Pages to OB/ 24 Hours
47,016	Systolic BP < 90	1170	2.49%	12.86	Systolic BP < 85	581	1.24%	6.38	Systolic BP < 85	87	0.956
47,016	Systolic BP > 160	910	1.94%	10.00	Systolic BP > = 160	1021	1.03%	11.22	Systolic BP > = 160	260	2.85
97,558	Diastolic BP > 100	1317	2.80%	14.47	Diastolic BP > = 110	483	1.24%	5.31	Diastolic BP > = 110	37	0.407
143,799	SpO2 < 95%	4996	5.12%	54.90							
143,799	Heart Rate < 50	602	0.42%	6.62							
47,016	Heart Rate > 120	4676	3.25%	51.38	Heart Rate > = 130	2168	4.61%	23.82	Heart Rate > = 130	190	2.09
	**Total Events Per 24 Hours**			150.2	**Total Events Per 24 Hours**			46.7	**Total Pages Per 24 Hours**		6.30

^a^The data in this table are based on the vital signs recorded by the bedside monitors in 34 high risk patient rooms over a 91 day period from April 1, 2017 to July 1, 2017

^b^These vital signs frequencies in this table are taken directly from the bedside monitor without nurse verification, and therefore likely contain some artefactual readings

Liberalizing the MEWC to less strict criteria, withholding pager alerts during stage two of labor, eliminating pages for hypoxemia and bradycardia, and utilizing the automated paging algorithm described above reduces the paging burden to the patient’s managing service dramatically. As shown in Table 3, the 34 rooms capable of automated vital signs capture generated 6.30 pages to the obstetrics team (OB) per day (one page every 3.8 h) from the original 150.2 readings per day (one reading every 9.2 min) that met the MEWC. By contrast, the use of automated pager alerts avoided the delays and errors inherent in manually generated alerts and was able to alert medical and nursing staff in a more timely fashion.

## Discussion

This maternal monitoring surveillance system was designed to provide clinicians with a consolidated overview of their labor and delivery service while also drawing attention to specific patients with high risk conditions and those experiencing changes in their clinical status.

Electronic patient surveillance systems have been deployed across a variety of patient care settings. Automated paging systems to detect severe sepsis have been used in the emergency department [11] and intensive care unit [12]. While no improvement was demonstrated in time to antibiotic administration, one study found an improvement in the time to draw blood cultures. Intraoperatively, AlertWatch™ OR has been shown to improve intraoperative glucose management [9], encourage ventilation of patients with a tidal volume of less than 10 mL/kg ideal body weight, and was associated with reduced hospital charges [14].

The software described in this manuscript has several unique features that have the potential to assist in managing a labor and delivery unit. Unlike previously described surveillance systems, this system provides a visualization of the labor and delivery unit. A rectangle represents each patient and is organized into a larger patient census depicting the entire labor and delivery service (Fig. 2). A color and letter coding system coincides with the coding from the institution’s inpatient electronic health record (MiChart, an implementation of the EPIC clinical information system, EPIC Systems Inc., Verona, WI). The census identifies antepartum, laboring, postpartum, and triage patients, as well as other patient conditions such as fetal loss or cesarean delivery. Laboring patients have cervical dilation displayed. Patient room numbers, body mass index, gravidy, parity and estimated gestational age are displayed in the same location of each patient rectangle. The bottom of each rectangle provides a quick summary of pertinent conditions (chosen via expert consensus) that affect a patient’s obstetric and anesthetic management using an icon system. Colored and flashing square icons, tied to the automated paging system, are also used to draw attention to specific patients that may be experiencing active clinical deterioration.

Any patient rectangle on the patient census can be selected for individual review. This individual patient display, is comprised of readily identifiable icons identifying different organ systems, whose colors change as measured parameters change from normal to marginal to profoundly abnormal ranges (Fig. 3). If one of the previously described alerts is present, it is prominently displayed in a column on the right side of the screen. Presentation of patient data in this format facilitates rapid assimilation of critical patient data. Additionally, visual and paging alerts may be used for separate indications to allow more frequent use of less intrusive visual alerts, and less frequent use of more intrusive paging alerts.

The multi-layered visual alerting system with black alerts, red alerts, and flashing red alerts combined with the tiered paging system, notifies providers of potentially dangerous changes in a patient’s condition. Automated alerts may improve patient safety by prompting providers to review and confirm abnormal vital signs. These alerts also remove cognitive bias that delays requests for patient evaluation and escalation of care by notifying managing services automatically. Healthcare providers on labor and delivery routinely share in one of the most joyful experiences in the lives of our patients and the majority of deliveries on an average labor and delivery unit are clinically unremarkable. It may therefore be easier for providers to ignore aberrant changes in maternal condition. Indeed, delays in diagnosis and treatment are a leading cause of maternal death [2, 3], and implementation of maternal early warning systems has been shown to decrease maternal morbidity [6]. We therefore believe that the novel application of this electronic surveillance system to the labor and delivery unit has the potential to yield benefits to patient care not identified in other settings [11, 12]. Anecdotally, the system has received support from nursing, obstetrics and anesthesiology staff as well as hospital administration; however, this support has not been confirmed objectively.

As this is a web-based system, providers can log into their unit via a secure Virtual Private Network and survey any patient from anywhere in the world. While there are logistical and medicolegal hurdles that must be overcome before remote monitoring on this scale is widely implemented, this feature may allow high risk centers to provide remote monitoring to other units affiliated with the hospital network.

In addition to its clinical application, this system offers research opportunities. For example, there is a remarkably high frequency of patient vital signs meeting the MEWC, shown in Table 3. If bedside monitors were programmed to automatically page a clinician for every vital sign meeting the MEWC, then the clinician managing these 34 beds would receive a page every 9.6 to 10.3 min. Even with the assumption that half of these vital signs are artefactual, this would still correspond to a high frequency of pages with vital signs meeting the MEWC every 19.2 to 20.6 min. By observing real time physiologic values that coincide with maternal pathology, it may be possible to better define the appropriate maternal early warning vital sign triggers. In addition, this surveillance system is being used in institutional studies and quality improvement projects to assess the utility of shock index in postpartum patients and to improve response time to severe hypertension.

This system continues to undergo iterative development, testing, and improvement based on feedback from anesthesiologists, obstetricians, nurse midwives, and bedside labor and delivery nurses. The alerts within the system, including the timing of the paging alerts, are customizable in future iterations. While this system was created as a “safety net,” one potential unintended consequence is that providers solely rely on the electronic surveillance system to notify them of a patient’s condition, rather than actively monitoring their patient. Additionally, little is known about the sequela of delaying pages to obstetricians and anesthesiologists while awaiting nurse confirmation of vital signs. While it is thought that this 10–30 min delay strikes an appropriate balance between delayed recognition of a dangerous change in the patient’s condition with preventing alarm fatigue, further investigation is needed.

Finally, it is unknown what paging frequency will lead to alarm fatigue. Automated pages sent to physicians were reduced by using the patient’s bedside nurse as a first-pass filter to verify aberrant vital signs. Given the large number of labor nurses (210 in our unit), the overall nursing paging burden is believed to be low. However, nurses will likely bear a higher frequency of pages when caring for more acutely ill patients. While an automated paging frequency of one page every 2.3 h is probably less likely to cause alarm fatigue than one automated page every 9.6 min, alarm fatigue is a complicated phenomena involving alarm severity, clinician workload, alarm frequency, and alarm accuracy, among other factors [15]. Only alarm frequency was considered in this manuscript. Prospective outcome studies will better inform on the impact of this surveillance system on patient outcome on the one hand and alarm fatigue on the other.

## Conclusions

Nationally, labor and delivery units continue to care for parturients with increasingly complex comorbidities. We describe AlertWatch™ OB, a novel electronic monitoring surveillance system, which is designed to visually summarize individual and aggregate patient information and alert staff of clinical deterioration. Prospective studies will be required to assess the impact on patient outcome.

## Abbreviations

AIMS: Anesthesia information management system
API: Application programming interface
BP: Blood pressure
EHR: Electronic health record
FDA: Food and Drug Administration
HL7: Health level 7
ICD: International Classification of Diseases
MEWC: Maternal Early Warning Criteria
OB: Obstetrics managing team
PACU: Post anesthesia care unit
RN: Registered Nurse
SpO2: Peripheral capillary oxygen saturation by pulse oximeter measurement
SQL: Structured query language
